# Enhanced Ocular Anti-Aspergillus Activity of Tolnaftate Employing Novel Cosolvent-Modified Spanlastics: Formulation, Statistical Optimization, Kill Kinetics, Ex Vivo Trans-Corneal Permeation, In Vivo Histopathological and Susceptibility Study

**DOI:** 10.3390/pharmaceutics14081746

**Published:** 2022-08-22

**Authors:** Diana Aziz, Sally A. Mohamed, Saadia Tayel, Amal Makhlouf

**Affiliations:** 1 Department of Pharmaceutics and Industrial Pharmacy, Faculty of Pharmacy, Cairo University, Cairo 11562, Egypt; 2 Department of Microbiology and Immunology, Faculty of Pharmacy, Cairo University, Cairo 12613, Egypt

**Keywords:** tolnaftate, spanlastics, cosolvent, fungal keratitis, kill kinetics, susceptibility

## Abstract

Tolnaftate (TOL) is a thiocarbamate fungicidal drug used topically in the form of creams and ointments. No ocular formulations of TOL are available for fungal keratitis (FK) treatment due to its poor water solubility and unique ocular barriers. Therefore, this study aimed at developing novel modified spanlastics by modulating spanlastics composition using different glycols for enhancing TOL ocular delivery. To achieve this goal, TOL basic spanlastics were prepared by ethanol injection method using a full 3^2^ factorial design. By applying the desirability function, the optimal formula (BS6) was selected and used as a nucleus for preparing and optimizing TOL-cosolvent spanlastics according to the full 3^1^.2^1^ factorial design. The optimal formula (MS6) was prepared using 30% propylene glycol and showed entrapment efficiency percent (EE%) of 66.10 ± 0.57%, particle size (PS) of 231.20 ± 0.141 nm, and zeta potential (ZP) of −32.15 ± 0.07 mV. MS6 was compared to BS6 and both nanovesicles significantly increased the corneal permeation potential of TOL than drug suspension. Additionally, in vivo histopathological experiment was accomplished and confirmed the tolerability of MS6 for ocular use. The fungal susceptibility testing using *Aspergillus niger* confirmed that MS6 displayed more durable growth inhibition than drug suspension. Therefore, MS6 can be a promising option for enhanced TOL ocular delivery.

## 1. Introduction

Fungal keratitis (FK) is one of the serious corneal infections that can cause eye damage and blindness if not treated effectively. Ocular trauma is considered the most common cause of FK as it can introduce fungi directly into the cornea [1]. It is evident that the most common causative organisms of FK are *Candida* and filamentous fungi (like *Fusarium species* and *Aspergillus species*) [2]. *Aspergillus* spp., if not diagnosed early, can result in macular involvement, damage of the choroid, and necrosis in the retina with subsequent reduction in visual capacity [3]. The spectrum of *Aspergillus* spp. is causing FK to become broader than previously believed [4]. Furthermore, *Aspergillus* is resistant to hot and dry conditions [5]. Hence, FK caused by *Aspergillus* spp. significantly increased over the last few years.

Tolnaftate (TOL) is a synthetic thiocarbamate antifungal agent that acts selectively against filamentous fungi, e.g., *Aspergillus* spp. Its fungicidal activity is mediated by inhibiting squalene epoxidase, which is an important enzyme in the biosynthesis of ergosterol (an important constituent of the fungal membrane). Therefore, squalene accumulates in the fungal plasma membrane and ergosterol is diminished resulting in negative effects on the membrane permeability and fungus growth resulting in cell death [6]. However, the conventionally available topical dosage forms of TOL, e.g., creams, gels, and ointments are of limited efficacy due to their poor penetration potential which requires long-term therapy and consequently decreases patient compliance [7]. Hence, several researchers have put forward their efforts in designing novel topical particulate carriers for enhancing the penetration of TOL for better therapeutic efficacy and increased patient compliance. These carriers include solid lipid nanoparticles [8], nanostructured lipid carriers [8], proniosomes [9,10], niosomal gels [11], and liposomal gels [7]. TOL is predictable to be a favorable therapeutic agent for the treatment of FK due to its selective fungicidal activity, lipophilic character (log P 5.5), and intermediate molecular weight (307.4). These physico-chemical properties enhance TOL permeability through the lipid-rich fungal cell membrane [12]. However, there is no satisfactory data in earlier literature about the use of TOL in an appropriate ocular delivery system for FK treatment due to its poor water solubility (0.00054 mg/mL) and the unique ocular barriers which consequently limit its ocular efficacy [13]. Our research team formulated TOL in novel polymeric pseudorotaxans and confirmed its superiority over TOL suspension in enhancing its ocular permeation and retention [14]. The challenge in this article is to formulate TOL in another novel delivery system for enhancing its water solubility with resultant enhancing its ocular permeation and retention.

The blinking reflex of the eye and its tear film turnover reduce the amount of the applied dose available to be absorbed. The remaining drug must then penetrate through the corneal epithelial constricted junction to be therapeutically effective [15]. Hence, to achieve satisfactory antifungal activity, an ideal topical drug delivery system should possess high corneal penetration, prolonged retention time with the eye, simplicity of installation, decreased frequency of administration with minimal side effects, and better patient compliance [16]. Accordingly, several novel drug delivery systems have been adopted for enhancing the ocular delivery of antifungal agents and improving their ocular bioavailability such as nanovesicles (niosomes, liposomes), elastic vesicles (transferosomes, spanlastics), microemulsions, solid lipid nanoparticles, and polymeric mixed micelles.

Spanlastics, span-based elastic vesicles, are referred to as modified niosomes. Like niosomes, spanlastics are uni/multilamellar spherical structures composed mainly of lipophilic non-ionic surfactant (span) with an additional edge activator that imparts flexibility to their walls [17]. Edge activators (single chain surfactants) can destabilize the vesicles and improve their vesicular bilayer deformability by lowering their surface tension [18]. Hence, the high elasticity of spanlastics helped them to enhance drug permeation through different mucosal bio-membranes (skin, cornea, gastrointestinal mucosa, etc.), by squeezing themselves through membrane pores with minimal risk of vesicular rupture [19]. Spanlastics had been investigated previously for their capability to augment the ocular, trans-duodenal, and transdermal drug absorption [17,20,21].

In the last two decades, great progress has been made in the field of formulating vesicular nano-carriers. For example, water was partially replaced with a cosolvent, e.g., ethanol, glycerol, and propylene glycol in order to induce a degree of elasticity to the vesicular bilayer [22]. In addition to enhancing the vesicular bilayer fluidity, these additives can enhance TOL water solubility due to their high solubilizing power [23]. It had been reported that the transdermal delivery of diclofenac and baicalin was enhanced via the incorporation of cosolvents in the vesicular constructs [22,24]. Up to date, the use of cosolvent-tailored nanovesicles for enhancing ocular delivery has not been yet investigated. Therefore, this research was conducted for accomplishing two main goals; the first one was to formulate TOL spanlastics according to 3^2^ factorial designs using Design-Expert^®^ software in order to study the effect of various formulation variables on the prepared spanlastics properties and to select the optimal formulation using desirability function. Secondly, to confirm the hypothesized capacity of cosolvents in enhancing the vesicular deformability, the optimal basic spanlastics were utilized as the nucleus for fashioning novel tailored spanlastics by modifying their composition using various cosolvents. Tailored spanlastics were prepared using a full 2^1^3^1^ factorial design for studying the effect of the type and proportion of the used cosolvent on the vesicular properties and selecting the optimal one based on the desirability function.

## 2. Materials and Methods

### 2.1. Materials

Tolnaftate (TOL) was supplied by Hikma pharmaceuticals (Cairo, Egypt). Span 60 (sorbitan monostearate) and Tween 80 (polyoxyethylene sorbitan monooleate) were acquired from Sigma Chemical Co. (St. Louis, MO, USA). Absolute methanol, absolute ethanol, glycerol, and propylene glycol were acquired from El-Nasr Pharmaceutical Chemicals Co. (Abu-Zaabal, Cairo, Egypt).

### 2.2. Preparation of TOL Basic Spanlastics

TOL basic spanlastics (prepared without the incorporation of cosolvents) were formulated using ethanol injection method [25]. Precisely, Span 60 and TOL (10 mg) were dissolved in 5 mL of absolute ethanol which was sonicated for 5 min at 80 °C to obtain a clear solution. The resultant alcoholic solution was then rapidly injected into the pre-heated aqueous solution (Tween 80, as edge activator, dissolved in 10 mL ultra-pure distilled water and heated to the temperature of 80 °C at 600 rpm using a 30 gauze syringe). The ratio of Span 60 to Tween 80 was optimized using Design-Expert^®^ software (Version 7, Stat-Ease, Inc., Minneapolis, MN, USA) according to full 3^2^ factorial design (Table 1). Post hoc analysis was performed using Tukey’s honest significant difference (HSD) test using SPSS software 17.0 (SPSS Inc., Chicago, IL, USA). Stirring was continued at 80 °C for 30 min. Spanlastics were formed spontaneously turning the solution slightly turbid. Then, the resultant turbid solution was left for another 30 min on a magnetic stirrer at room temperature for ethanol removal by evaporation. For reduction of PS, the prepared formulae were sonicated in a bath sonicator at 25 °C for 10 min. TOL basic spanlastics formulations were then left overnight to equilibrate at 4 °C.

### 2.3. In Vitro TOL Basic Spanlastics Characterization

#### 2.3.1. Entrapment Efficiency (EE%)

TOL basic spanlastics formulae were filtered using Whatman filter paper (grade No. 1, 11 μm) to separate the unentrapped drug due to the extremely low solubility of TOL in water [17,26,27] whereas vesicles loaded with TOL passed through the filter paper to the filtrate. Then, 0.3 mL of the filtrate were then sonicated with methanol, and the entrapped TOL concentration was measured spectrophotometrically at 257 nm. Each result was expressed as the mean of three measurements ±SD. TOL EE% was calculated by the following formula:

$$\mathrm{EE}\%=\frac{\mathrm{Incorporated}\;\mathrm{amount}\;\mathrm{of}\;\mathrm{TOL}}{\mathrm{Totlal}\;\mathrm{amount}\;\mathrm{of}\;\mathrm{TOL}}\times 100$$

#### 2.3.2. Particle Size (PS), Polydispersity Index (PDI), and Zeta Potential (ZP)

Zetasizer Nano ZS (Malvern Instrument Ltd., Worcestershire, UK) was used in the determination of PS, PDI, and ZP of the prepared TOL basic spanlastics applying dynamic light scattering technique. The formulations were diluted with deionized water prior to measurement to produce proper scattering intensity. Each measurement was carried out in triplicates.

### 2.4. TOL Basic Spanlastics Formulation Optimization

Desirability function was applied to select the optimum TOL basic spanlastics. The optimization process targeted to achieve a formula with the highest EE% and ZP (as absolute value) and the least PS as presented in Table 1. The formula with the highest desirability value (near to one) was chosen. To check whether the responses predicted by the software are valid or not, the optimized TOL basic spanlastics formula was prepared and characterized and its responses were compared to the predicted ones [28].

### 2.5. Modification of the Optimal TOL Basic Spanlastics Using Cosolvents

Cosolvent-modified spanlastics (TOL-cosolvent spanlastics) were prepared using the same components of the optimal basic spanlastics together with different cosolvents (glycerol and propylene glycol in different concentrations). Formulations were prepared using the same ethanol injection technique employed for the preparation of the basic spanlastics, and the cosolvent was added to the pre-heated aqueous medium. 

### 2.6. Statistical Design for the Preparation of TOL-Cosolvent Spanlastics

A full factorial design (3^1^.2^1^) was utilized using Design-Expert^®^ software to prepare TOL-cosolvent spanlastics. Changing the utilized cosolvent (X_1_) and its percentage (%*v*/*v*) (X_2_) were considered as the independent variables X_1_ and X_2_, respectively. On the other hand, EE% (Y_1_), PS (Y_2_), and ZP (Y_3_) were the selected dependent variables as shown in Table 2. For the percentage of the used cosolvent (X_2_), post hoc analysis was performed by Tukey’s HSD test utilizing SPSS software 17.0.

### 2.7. In Vitro TOL-Cosolvent Spanlastics Characterization

#### 2.7.1. Entrapment Efficiency (EE%)

EE% of TOL-cosolvent spanlastics was determined using the same procedures followed for determination of EE% of TOL spanlastics.

#### 2.7.2. Particle Size (PS), Polydispersity Index (PDI), and Zeta Potential (ZP)

As previously mentioned, PS, ZP, and PDI were determined using Zetasizer at 25 °C.

### 2.8. TOL-Cosolvent Spanlastics Optimization

The optimum TOL-cosolvent spanlastics composition was predicted by applying the desirability function method using Design-Expert^®^ software (Version 7, Stat-Ease, Inc., Minneapolis, MN, USA). The optimization criteria were the smallest PS, the highest EE%, and absolute value of ZP (Table 2). The formulation with the highest desirability value was chosen for further characterizations. After selection, the optimal formulation was prepared, characterized, and compared with the predicted responses to confirm the model efficacy. Thereafter, to explore the effect of the added cosolvent on the vesicular physico-chemical properties, the characteristics of the optimal cosolvent tailored spanlastics (PS, ZP, and EE%) were compared to those of the optimal basic spanlastics. Student’s *t*-test was used to statistically analyze the results.

### 2.9. Transmission Electron Microscopy (TEM)

The morphological features of the selected optimal TOL-cosolvent spanlastics were assessed using TEM (Joel JEM 1230, Tokyo, Japan). One drop of the undiluted sample was placed on a carbon-coated copper grid and allowed to dry for about 10 min at room temperature and subsequently investigated. 

### 2.10. Differential Scanning Calorimetry (DSC)

Thermal analysis of TOL, Span 60, the optimum TOL-cosolvent spanlastics formula, and the physical mixture of TOL with spanlastics ingredients was accomplished by a previously calibrated differential scanning calorimeter (DSC-60, Shimadzu, Kyoto, Japan). Each sample (≈5 mg) was placed in a standard aluminum pan and heated in a temperature range of 10–300 °C at a heating rate of 10 °C/min with continuous purging of nitrogen (25 mL/min). 

### 2.11. Ex Vivo Studies

#### 2.11.1. Corneas Preparation

The Research Ethics Committee of the Faculty of Pharmacy, Cairo University, Egypt approved the study (PI 2982). Adult male New Zealand albino rabbits (2.5–3.0 kg) were used to extract the corneas. The rabbits were decapitated following anesthesia with an intramuscular injection of ketamine 35 mg/kg, and a relaxing agent: xylazine 5 mg/kg [29]. The eyes were enucleated immediately, and the corneas were excised and cautiously cleaned with saline and checked for being free from any pores before using in the test. The permeation experiment was conducted within half an hour of corneas extraction [25]. 

#### 2.11.2. Corneal Permeation Study

A modified static Franz diffusion cell (area = 0.64) was used in TOL corneal permeation studies. The excised corneas were sandwiched between the donor and receptor chamber. The receptor medium was 100 mL of methanol: water (3:2), and it was kept under continuous stirring (100 rpm) at 37 °C [30]. Accurate volume of each of the optimal TOL-cosolvent spanlastics, TOL basic spanlastics, and TOL aqueous suspension (equivalent to 300 µg TOL) was loaded under non-occlusive conditions on the corneal surface (donor compartment). At different time intervals (0.5, 1, 2, 4, 6, and 8 h), 3 mL volumes were withdrawn from the receptor compartment and fresh medium was added to replace the withdrawn samples. The study was performed three times and the results were presented as mean ± SD. TOL amount in the samples was determined by a validated HPLC system (Shimadzu, Kyoto, Japan) equipped with L-7110 pump unit and an X Terra ^TM^ column (Reversed C18:4.6 mm × 250 mm) having 5 µm size adsorbent as stationary phase (Milford, CT, USA). We used methanol 80% (*v*/*v*) as a mobile phase which flowed with a rate of 1.2 mL/min, and the drug was detected at 258 nm [31]. The assay procedures were validated for linearity, accuracy, and precision.

The cumulative amount of TOL permeated per unit area (µg/cm^2^) was plotted against time (h). The flux (J_max_) at 8 h and the enhancement ratio (ER) were calculated using the following equations [32].

$$J_{\max}=\frac{\mathrm{Amount}\;\mathrm{of}\;\mathrm{drug}\;\mathrm{permeated}}{\mathrm{Time}\times \mathrm{Area}\;\mathrm{of}\;\mathrm{membrane}}$$

$$\mathrm{ER}=\frac{J_{\max}\mathrm{of}\;\mathrm{the}\;\mathrm{optimal}\;\mathrm{nanoformulation}}{J_{\max}\mathrm{of}\;\mathrm{the}\;\mathrm{drug}\;\mathrm{suspension}}$$

The differences in J_max_ and total TOL permeated were statistically analyzed using SPSS software 17.0. Post hoc analysis was performed using Tukey’s HSD test. The difference was considered significant when *p* ≤ 0.05.

### 2.12. In Vitro Antifungal Activity

#### 2.12.1. Fungal Strain and Inoculum Preparation

In this study, *Aspergillus niger* standard strain (ATCC32656) was tested. Sabouraud Dextrose Agar (SDA) (Oxoid, Hampshire, UK) was used as the growth medium and the plates were incubated for 48–96 h at 28 ± 2 °C. The germinating spores were harvested in sterile normal saline solution, and the inoculum size was adjusted to 10^5^–10^6^ CFU/mL count.

#### 2.12.2. Tested Samples

For the in vitro antifungal activity, the tested treatments were (i) the optimum TOL-cosolvent spanlastics (MS6) (treatment A), (ii) TOL aqueous suspension (1 mg/mL) (treatment B), and (iii) placebo solution (TOL-free optimum cosolvent-spanlastics formula).

#### 2.12.3. Minimum Inhibitory Concentration (MIC)

The determination of MIC was performed utilizing microbroth dilution technique according to Sayed et al. [30,33]. Two-fold serially diluted treatments A and B were set in double strength Sabouraud Dextrose Broth (SDB) (500–0.24 µg/mL), dispensed into U-shaped bottom 96-well plates, then 10 μL of the spore suspension (inoculum size of 10^5^–10^6^ CFU/mL) was added to each well. Negative control (double strength SDB only) and positive control (double strength SDB and two-fold serial dilution of placebo solution with 10 μL of the inoculum) were adopted. The microplates were incubated at 28 ± 2 °C for 48 h. The MIC was determined as the lowest concentration having no observable fungal growth. Triplicates of the experiment were conducted.

#### 2.12.4. Minimum Fungicidal Concentration (MFC)

For treatment A and treatment B, the determination of MFC was performed using broth microdilution method according the Clinical and Laboratory Standards Institute guidelines [33]. Briefly, 2-fold serially diluted treatment A or B till the MIC together with the 10 μL spore suspension at 28 ± 2 °C were incubated in 96-well plates for 24 h. Then, 10 µL of mixture was spotted on SDA plates. The plates were then incubated at 28 ± 2 °C for 48 h then the fungal colony count (as CFU/mL) was determined. The MFC was expressed as the lowest treatment concentration that shows no fungal growth. 

#### 2.12.5. Kill Kinetics Assay

Time–kill kinetics of treatment A and treatment B against *Aspergillus niger* (ATCC32656) were conducted according to the method described by Ismail et al., with some modifications [34]. Briefly, the killing kinetics of treatment A and treatment B were assayed at the fungicidal concentrations. In a 96-well microtiter plate, 100 µL of the double strength SDB and 100 µL of treatment A or treatment B in its MFC concentration together with 10 µL of the spore suspension (inoculum size of 10^5^–10^6^ CFU/mL). Ten microliters of this mixture were added to 90 µL of saline solution, 10-fold serially diluted, and subjected to viable colony count on SDA medium (0 time sample). The 96-well microtiter plate containing the mixture of treatment and fungal spores was incubated at 28 ± 2 °C for up to 24 h. Then, samples of each test were withdrawn at time intervals of 4, 6, 10, 16, and 24 h, diluted, and subjected to viable colony count on SDA medium. The test was performed in triplicates.

### 2.13. In Vivo Studies

#### 2.13.1. Animals

The Research Ethics Committee at Faculty of Pharmacy, Cairo University, Egypt (PI 2982) approved the study. Twelve male albino rabbits (2–3 kg) were used in the study. The rabbits were individually caged under proper conditions of humidity, temperature (25 ± 2 °C), and 12 h light/dark cycles. The animals were on a standard dry food and water ad libitum. Examination of the rabbits by a slit lamp was performed to exclude animals with ocular inflammation or disorder before the study.

#### 2.13.2. Draize Test

In order to assess the irritation potential of the optimal formula, a scoring system was applied using three male albino New Zealand rabbits [29]. An aliquot of 100 µL of the optimal TOL-cosolvent spanlastics was added to the right eye (in the conjunctival sac) and the left eye was treated with normal saline to serve as control. We examined the right eye visually at 1, 2, 5, 8, and 24 h after installation for any irritation, and the eye was scored according to Draize scale [19,29]. The Draize scale was given as follows: 0, no reaction; 1, very slight erythema; 2, well-defined erythema; 3, moderate to severe erythema [19].

#### 2.13.3. Histopathology

Male albino New Zealand rabbits (3 animals) were utilized to assess the safety of the prepared spanlastics. The optimum TOL-cosolvent spanlastics (one drop) were dropped into the rabbit’s right eye and normal saline was installed into the left eye as control. The treatments were repeated at one-hour intervals for a total of six hours [35]. Then, the rabbits were anesthetized and euthanized and their eyeballs were extracted, washed with normal saline solution, and fixed in 10% formalin in saline for 24 h. Then, the samples were washed with tap water followed by serial dilutions of alcohols to dehydrate the samples. Then, specimens were cleared in xylene and fixed in paraffin for 24 h at 56 degrees in hot air oven. A sledge microtome (Leica Microsystems SM2400, Cambridge, UK) was used to prepare paraffin beeswax tissue blocks by sectioning at 4 microns thickness. Then tissue sections were presented on glass slides, deparaffinized, stained by hematoxylin and eosin stain to be examined under light electric microscope [36].

#### 2.13.4. Susceptibility Test

The tested microorganism was *Aspergillus niger* (ATCC32656). A parallel design of two groups each composed of three rabbits was applied. The study was performed according to Albash et al. [37]. Group I was administered the optimal TOL-cosolvent spanlastics (treatment A) and group II was administered TOL suspension (treatment B). Equal volumes (50 µL containing 50 µg TOL) of treatment A and treatment B were applied in the lower conjunctival sac of the rabbit’s right eye using micropipette. No treatment was applied in the left eye of all rabbits to serve as the control. At predetermined intervals (2–24 h), four sterile filter paper discs (Whatman no. 5, 6 mm in diameter) were wetted by placing the discs under the eyelid of each eye. For each eye (right and left), two discs were placed in a 1.5 mL Eppendorf tube containing 500 µL SDB inoculated with 10% *v*/*v* fungal spore suspension (10^5^–10^6^ CFU/mL). The other two discs were placed in a 1.5 mL Eppendorf tube containing 500 µL uninoculated SDB as a blank for measuring the optical densities. All tubes were then incubated at 28 ± 2 °C for 48 h under aerobic conditions. At the end of the incubation period, 200 µL of each tube was transferred to a sterile 96-well plate and the optical densities (OD_600 nm_) were read on an automated spectrophotometric plate reader (Biotek, Synergy 2, Winooski, VT, USA). The results were presented as growth inhibition % calculated according to the following equation:

$$\mathrm{Growth}\;\mathrm{inhibition}\;\%=\frac{\mathrm{Control}\;\left(\mathrm{left}\;\mathrm{eye}\right)\;\mathrm{OD}600\;\mathrm{nm}\;-\;\mathrm{Test}\;\left(\mathrm{right}\;\mathrm{eye}\right)\;\mathrm{OD}600\;\mathrm{nm}\;}{\mathrm{Control}\;\left(\mathrm{left}\;\mathrm{eye}\right)\;\mathrm{OD}600\;\mathrm{nm}}\times 100$$

## 3. Results and Discussion

### 3.1. Preparation of TOL Basic Spanlastics

Based on the preliminary studies, the ethanol injection method was the most appropriate method for preparing TOL spanlastics. Span 60, a lipophilic non-ionic surfactant (HLB = 4.7), was selected due to the lipophilicity of its saturated alkyl chain which cards the formation of multi-lamellar vesicles [21]. Furthermore, the surface-active nature of Span 60 would boost the action of the edge activator in reducing the interfacial tension with the subsequent production of fine spanlastics dispersion. In contrast, it was shown that Span 40 and Span 80 formed vesicles with a high degree of instability and aggregation [38]. The incorporation of edge activators would enhance the vesicular elastic nature and increase their deformability. Different edge activators were used for preparing TOL basic spanlastics in the preliminary trials including Tween 80, Cremophor EL, and sodium cholate. Vesicles formed using Tween 80 as an edge activator showed better results with respect to EE% and PS (data not shown) and therefore, it was used in this study. Ethanol was shown to have a positive impact on these nanovesicular properties via enhancing the drug entrapping and partitioning within their vesicular bilayer [39]. Furthermore, ethanol can decrease vesicular size via different mechanisms. Firstly, it can reduce the thickness of the vesicular membrane due to its membrane condensing ability. Secondly, ethanol can modify the system net charge towards negative ZP with resultant steric stabilization [40]. As previously mentioned, sonication is necessary for developing fine dispersion with suitable PS. However, preliminary screening showed that there was an inverse relationship between EE% of the prepared vesicles and sonication time. Hence, sonication time was fixed to be 10 min which fashioned vesicles with optimum PS without significantly affecting PS or which achieved a good compromise between PS and EE%. 

### 3.2. Factorial Design Analysis of TOL Basic Spanlastics

A total of nine formulations of TOL basic spanlastics were prepared according to 3^2^ full factorial design which was statistically analyzed by Design-Expert^®^ software in order to analyze the combined effect of the selected variables on spanlastics’ properties. The selection of the levels of the factors was according to preliminary trials and the possibility of TOL spanlastics preparation utilizing these levels. Two-factor interaction (2FI) was the selected model. The measured responses of the nine experimental runs are presented in Table 3. The predicted R^2^ was calculated as a measure of the model efficiency in predicting a response value [6]. Referring to the results of design analysis (Table 4), it is worthy to note that the predicted R^2^ values was close to the adjusted R^2^ in all responses, which confirmed that the selected model adequately fitted to the data [41]. Adequate precision denotes the signal-to-noise ratio to confirm that the model can assess the design space [42]. A ratio greater than 4 is desirable, which was observed in all of the measured response (Table 4).

#### 3.2.1. Formulation Variables Effect on EE% of TOL Basic Spanlastics

**Figure 1:**
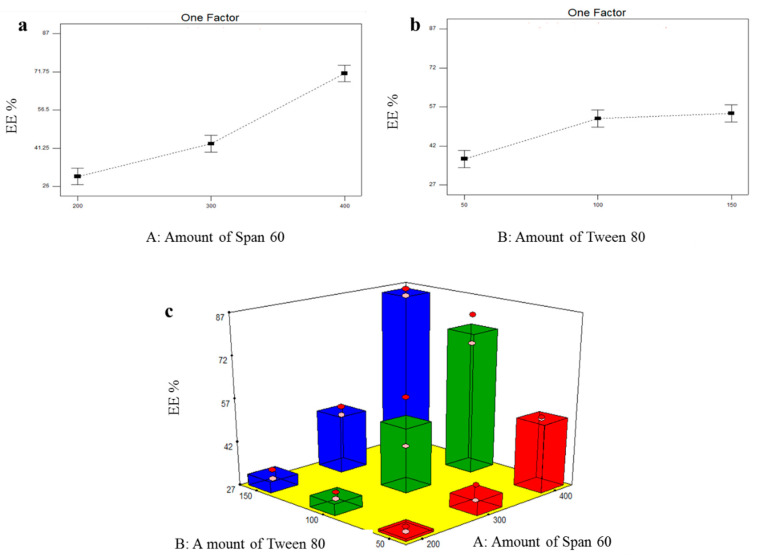
Line plots of the significant effect of Span 60 amount (X_1_) (**a**), Tween 80 amount (X_2_) (**b**), response 3D plot for the combined effect of amount of Span 60 (X_1_) and amount of Tween 80 (X_2_) (**c**) on EE% of TOL basic spanlastics.

The formulated basic spanlastics showed EE% ranging from 27.85 ± 1.20 (BS1) to 85.45 ± 1.77 (BS9) as shown in Table 3. The influence of the amount of Span 60 (X_1_) and the amount of Tween 80 (X_2_) on EE% of TOL basic spanlastics was graphically illustrated in Figure 1. X_1_ and X_2_ were shown to have significant effect on EE% (*p* ˂ 0.0001 and =0.0004, respectively) (Table 4). Post hoc analysis showed that spanlastics prepared using 400 mg Span 60 had the highest EE% compared to those prepared using 300 mg Span 60 and then those prepared using 200 mg Span 60 (*p* < 0.05). This might be related to increasing the amount of vesicle-forming material (Span 60), which might result in the formation of greater lipophilic ambiance to incorporate a higher amount of the hydrophobic drug (TOL) with the consequent increase in EE% [43]. With respect to the effect of using different amounts of Tween 80 (X_2_), post hoc analysis revealed that spanlastics prepared using 100 mg and 150 mg Tween 80 showed higher EE% compared to those prepared using 50 mg. The positive impact of edge activator amount (Tween 80) on EE% could be explained by the aptitude of edge activator, when used in optimum concentration, to endow more space for holding extra drug [44]. It could also be related to the capability of the edge activator monomolecular layer to create a coat over the composed spanlastics that can stabilize them. This coat is also able to hold more drugs and subsequently enhance EE% [45]. These findings agreed with that presented by El Menshawe et al. who showed that EE% of fluvastatin-loaded spanlastics increased by increasing edge activator concentration from 10% *W/W* to 20% *W/W* [44].

#### 3.2.2. Formulation Variables Effect on PS of TOL Basic Spanlastics

**Figure 2:**
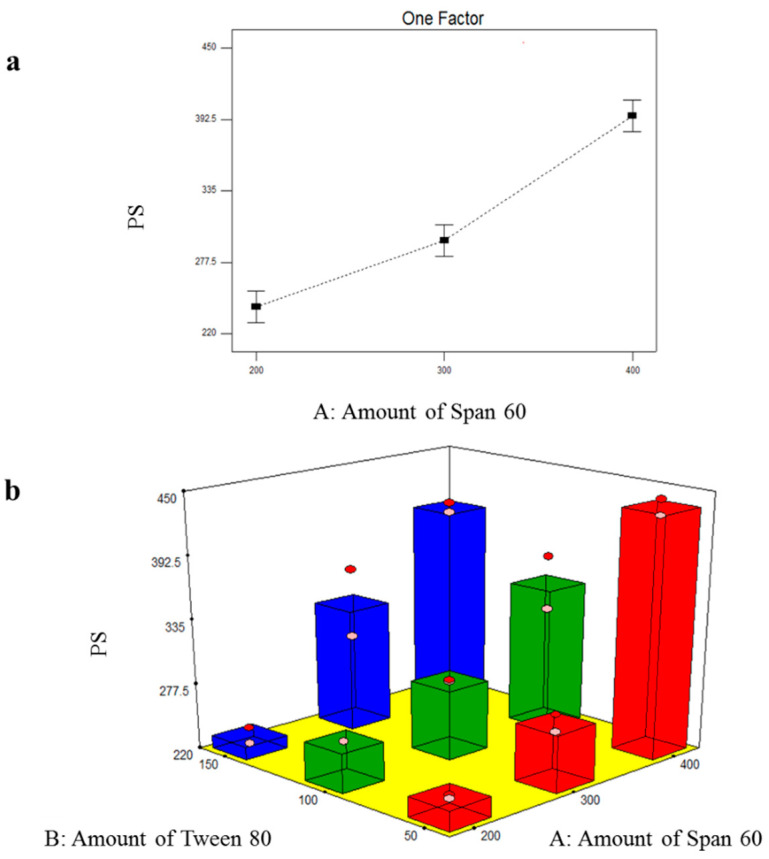
Line plot of the significant effect of Span 60 amount (X_1_) (**a**) and response 3D plot for the combined effect of Span 60 amount (X_1_) and Tween 80 amount (X_2_) (**b**) on PS of TOL basic spanlastics.

The particle size of the prepared TOL basic spanlastics ranged from 231.50 ± 10.18 nm to 436.55 ± 9.97 nm as shown in Table 3. The small PS plays a specific role in the safe and efficient ocular drug delivery. The influence of the amount of Span 60 (X_1_) and the amount of Tween 80 (X_2_) on PS of TOL basic spanlastics was graphically illustrated in Figure 2. ANOVA results revealed that only the amount of Span 60 (X_1_) significantly affected the PS of the prepared basic spanlastics (*p* < 0.0001) (Table 4). By increasing the amount of Span 60 (film-forming material), multiple layers would have accumulated over each other and consequently, PS increased. These results can also be correlated with the noticeable increase in EE% by increasing the amount of Span 60. As previously mentioned, increasing Span 60 amount increased the amount of TOL incorporated in vesicles’ hydrophobic region and consequently increased the distance between the vesicular lipid bilayer with the resultant increase in PS [43]. Oppositely, the PS of TOL basic spanlastics was not significantly affected by the amount of Tween 80 (X_2_) (*p* = 0.1270). 

#### 3.2.3. Formulation Variables Effect on ZP of TOL Basic Spanlastics

**Figure 3:**
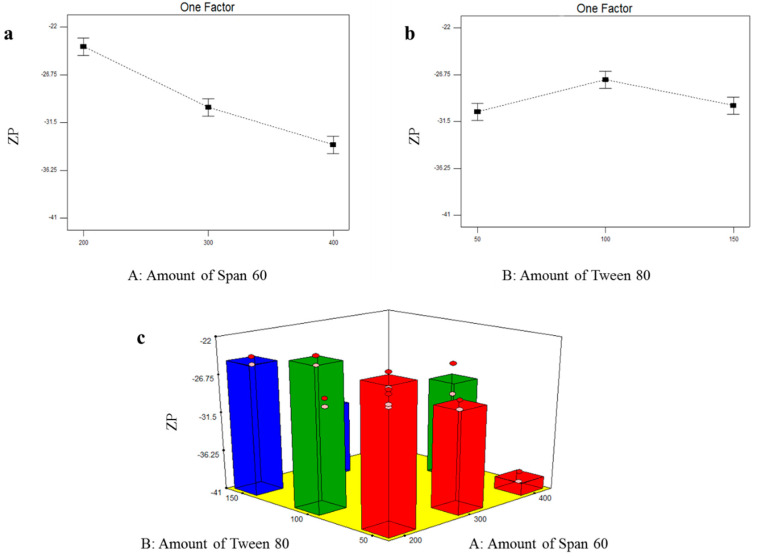
Line plots of the significant effect of Span 60 amount (X_1_) (**a**), Tween 80 amount (X_2_) (**b**), response 3D plot for the combined effect of Span 60 amount (X_1_) and Tween 80 amount (X_2_) (**c**), on ZP of TOL basic spanlastics.

The nanosystem stability is related directly to the magnitude of the electric charge adsorbed on its surface. It was observed that TOL basic spanlastics were with negative ZP values which fluctuated from −23.25 ± 0.78 to −39.45 ± 0.92 mV (Table 3). This negative charge will prevent aggregation and give more stable dispersion. The influence of the amount of Span 60 (X_1_) and the amount of Tween 80 (X_2_) on the ZP of TOL basic spanlastics is illustrated in Figure 3. Statistical analysis showed that both X_1_: the amount of Span 60 and X_2_: the amount of Tween 80 had a significant effect on ZP of TOL basic spanlastics (*p* ˂ 0.0001 and = 0.0047, respectively) (Table 4). With respect to the effect of the amount of Span 60 (X_1_) on ZP, post hoc analysis revealed that spanlastics prepared using 400 mg Span 60 had the highest ZP and that ZP increased by increasing the amount of Span 60 in a concentration-dependent manner. This might be attributed to the ionizable carboxylate group present within the polar head of Span 60, which is normally directed towards the aqueous external phase creating net negative ZP [46]. Hence, by increasing Span 60 amount, more negative carboxylate groups would reside on the vesicular surface, and consequently, ZP increases. With respect to the effect of the amount of Tween 80 (X_2_) on ZP of TOL basic spanlastics, the post hoc test showed that spanlastics containing 100 mg Tween 80 possessed the highest ZP compared to spanlastics prepared using 50 and 150 mg. The higher ZP values of spanlastics containing 100 mg compared to those of 50 mg are referred to as the EE% which increased when the Tween 80 amount increased as previously stated. TOL, containing an ionizable thiocarbamate group, could ionize and obtain a negative charge in alkaline and neutral pH. Hence, by increasing the amount of Tween 80 from 50 to 100 mg, more TOL would be entrapped within the vesicles with further ionization of the thiocarbamate group and consequently increased negative charge acquired by the formed spanlastics [47]. As previously mentioned, there was no significant difference in EE% of spanlastics containing 100 mg and those of 150 mg Tween 80. However, the latter showed significantly lower ZP values. Tween 80, being a non-ionic surfactant, can reside on the surface of the vesicles’ bilayer due to its hydrophilicity and consequently shield the acquired negative surface charge [48]. Hence, by increasing the amount of Tween 80, the negative ZP values gradually decreased due to the accumulation of the hydrophilic non-ionic surfactant (Tween 80) on the vesicular bilayer surface with consequent shielding of the surface negative charge [20,21].

### 3.3. Selection of the Optimal TOL Basic Spanlastics

In order to select the optimal formula from the nine prepared spanlastics formulations, a response surface analysis of the factorial design was performed using Design-Expert^®^ software. The predetermined constraints for optimization (minimizing PS and maximizing EE% and ZP, as absolute value), were achieved in BS6 with total desirability of 0.633. BS6 was composed of 400 mg Span 60 and 100 mg Tween 80 and showed EE% of 76.80 ± 7.07%, PS of 349.55 ± 34.44 nm, and ZP of −28.75 ± 2.76 mV. Therefore, BS6 was selected as a nucleus formula for preparing and optimizing novel cosolvent-modified spanlastics. 

### 3.4. Factorial Design Analysis of TOL-Cosolvent Spanlastics

To assess the effect of using different cosolvents on the physicochemical properties of spanlastics and to evaluate the effect of cosolvent amount on these properties, a full 3^1^.2^1^ factorial design was utilized and statistically analyzed through Design-Expert^®^ software. The model selected was 2FI. The measured responses of the six experimental runs are presented in Table 5. As shown in Table 6, adequate precision is greater than 4 (the desirable value) in all responses except ZP which was a non-significant model term. It was also noted that the predicted R^2^ values came in reasonable agreement with the adjusted R^2^ in all responses except ZP (Table 6). The negative predicted R^2^ value of ZP indicates that the overall mean is a better predictor of the response [49]. This might be due to the fact that the ZP of the prepared tailored spanlastics was not significantly influenced by any of the tested factors.

#### 3.4.1. Effect of Formulation Variables on EE% of TOL-Cosolvent Spanlastics

**Figure 4:**
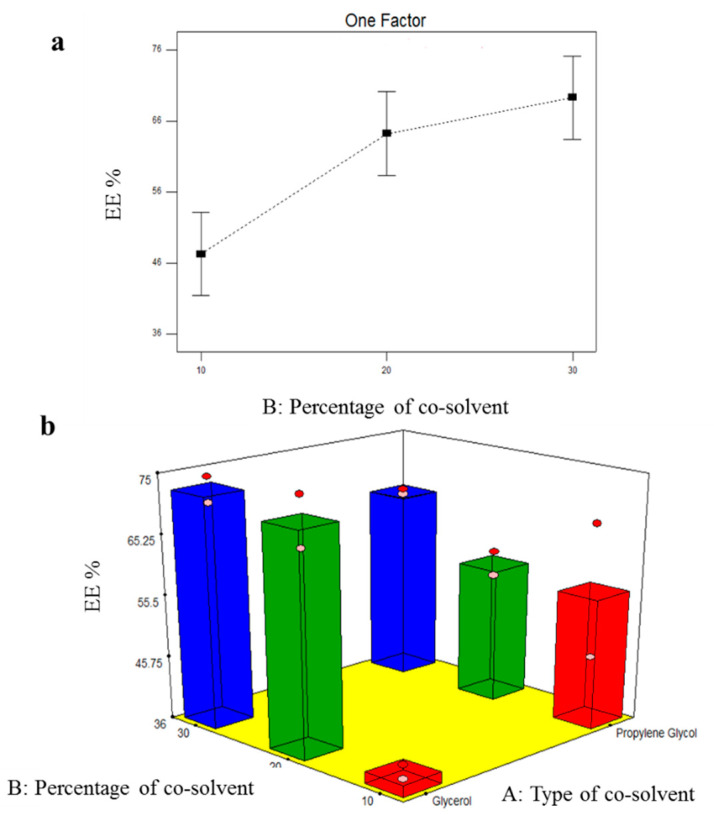
Line plots of the significant effect of cosolvent percentage (X_2_) (**a**) and response 3D plot for the combined effect of cosolvent type (X_1_) and cosolvent percentage (X_2_) (**b**), on EE% of TOL-cosolvent spanlastics.

EE% of all TOL-cosolvent spanlastics ranged from 37.80 ± 1.56 to 72.50 ± 2.83% as demonstrated in Table 5. The influence of type of cosolvent (X_1_) and percentage of cosolvent (X_2_) is graphically illustrated in Figure 4. Results of the ANOVA test revealed that type of cosolvent (X_1_) did not significantly affected EE% of TOL-modified spanlastics (*p* = 0.9339). On the other side, the percentage of the cosolvent (X_2_) possessed significant effect on EE% (*p* = 0.0089). By increasing the percentage of cosolvent (glycerol or propylene glycol), EE% significantly increased. This could be attributed to the cosolvent positive effect on TOL solubility in the lipid phase [50]. Furthermore, the higher concentration of cosolvent allowed a superior lipid packing and more efficient drug loading [51]. 

#### 3.4.2. Formulation Variables Effect on PS of TOL-Cosolvent Spanlastics

**Figure 5:**
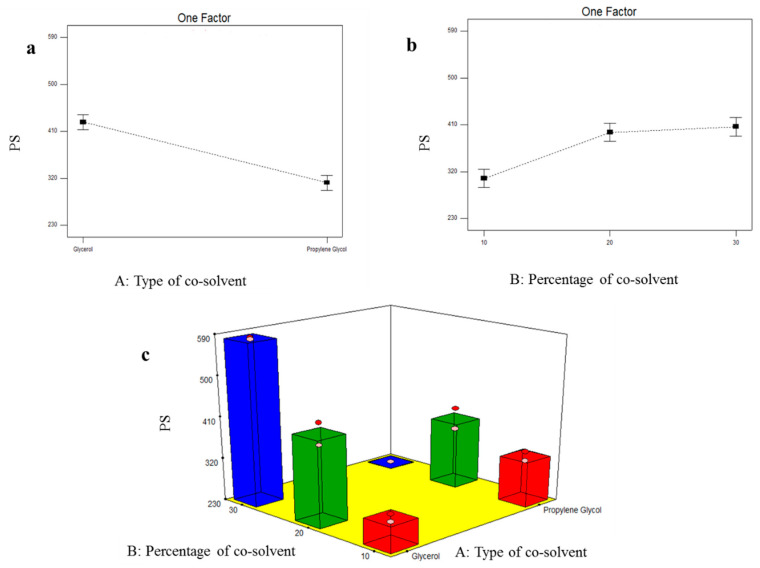
Line plots of the significant effect of type of cosolvent (X_1_) (**a**), percentage of cosolvent (X_2_) (**b**), response 3D plot for the combined effect of type of cosolvent (X_1_), and percentage of cosolvent (X_2_) (**c**) on PS of TOL-cosolvent spanlastics.

As shown in Table 5, the PS of the prepared TOL-cosolvent spanlastics fluctuated from 231.20 ± 0.141 to 581.50 ± 2.55 nm. Figure 5 illustrates the effect of type of cosolvent (X_1_) and percentage of cosolvent (X_2_) on PS of the prepared tailored spanlastics. Statistical analysis showed that type of cosolvent (X_1_) had a significant effect on PS (*p* ˂ 0.0001). Due to the higher viscosity of glycerol, it was shown that it formed significantly larger vesicles compared to propylene glycol-modified spanlastics [52]. Additionally, Manconi et al. reported the reduction in PS by incorporation of glycols [51]. It was also shown that the percentage of cosolvent (X_2_) significantly affected the PS of the prepared spanlastics (*p* = 0.0009). With respect to glycerol-based vesicles, the ANOVA test revealed that increasing concentration of glycerol caused the formation of bigger vesicles due to the glycerol viscous nature [52]. In contrast, 30% propylene glycol-based vesicles showed the smallest PS. This could be related to that the incorporation of propylene glycol caused reduction in vesicular size due to its interaction with vesicular bilayer causing more bilayer flexibility and small vesicle size. Hence, increasing propylene glycol percentage showed significant negative effect on vesicular size [50]. These results also came in agreement with Manconi et al. who reported that diclofenac-loaded vesicles showed significant decrease in PS by increasing the percentage of propylene glycol to 40 and 50% [51]. 

#### 3.4.3. Formulation Variables Effect on ZP of TOL-Cosolvent Spanlastics

The ZP of TOL-cosolvent spanlastics fluctuated between −28.70 ± 2.12 and −32.15 ± 0.07 mV as shown in Table 5. The ZP of the prepared spanlastics was not affected significantly by the studied factors (*p* > 0.05). 

### 3.5. Selection of the Optimal TOL-Cosolvent Spanlastics

Desirability was determined by identifying the optimal formula that has the highest EE% and ZP, as the absolute value, and the lowest PS. These desirability constraints were established in MS6 with overall desirability of 0.895. MS6 was prepared using 30% propylene glycol and showed EE% of 66.10 ± 0.57%, PS of 231.20 ± 0.141 nm, and −32.15 ± 0.07 mV. By comparing the measured responses for both BS6 and MS6, MS6 showed significantly smaller PS compared to BS6 (*p* = 0.04) due to the influence of the incorporated cosolvent (propylene glycol) on enhancing the vesicular bilayer flexibility and consequently producing vesicles with smaller PS [50]. Hence, MS6 was selected for further characterization. Oppositely both vesicles showed was no significant difference in EE% and ZP (*p* = 0.167 and 0.223, respectively).

### 3.6. Transmission Electron Microscopy (TEM)

**Figure 6:**
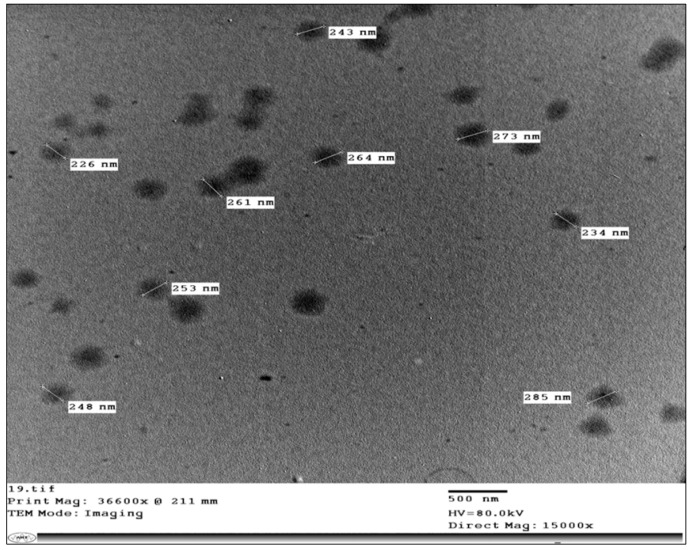
Transmission electron micrograph of MS6.

TEM imaging is valuable for describing the morphological features of the prepared system [41]. Illustrative photomicrographs of MS6 are exemplified in Figure 6. The developed modified spanlastics were well scattered without any aggregations. Furthermore, the vesicular diameter observed by TEM was in line with that previously measured using a Zetasizer and ranged from 200–300 nm.

### 3.7. Differential Scanning Calorimetry (DSC)

**Figure 7:**
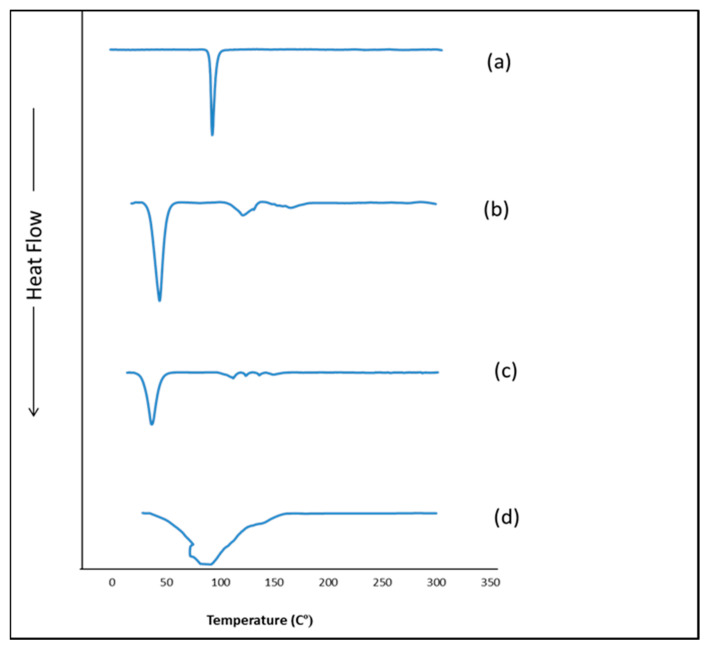
DSC thermograms of (**a**) TOL, (**b**) Span 60, (**c**) physical mixture of TOL-cosolvent spanlastics components, and (**d**) formula MS6.

The DSC thermograms of TOL, Span 60, TOL-Span 60-Tween 80-propylene glycol physical mixture, and MS6 are shown in Figure 7. Since the measuring temperature of DSC equipment ranged from 25 to 725 °C, the thermotropic properties of Tween 80 and propylene glycol, being liquids at room temperature, could not be assessed in this work [17]. The DSC thermogram of pure TOL showed a single endothermic peak at 112.31 °C corresponding to its melting point due to the TOL crystallinity [53]. The endothermic peak at 55.05 °C was for Span 60 and corresponded to its melting point [54]. Regarding the DSC thermogram of the physical mixture of TOL with cosolvent-modified spanlastics components, it showed the endothermic transition of TOL but with lower intensity compared to the pure drug. This might be attributed to the dilution of the drug with the used excipients [55]. Oppositely, the TOL endothermic peak was completely absent in the thermogram of MS6 confirming that TOL was entrapped in cosolvent-modified spanlastics and perfectly interacted with the vesicular bilayer. Furthermore, the development of a less-ordered lattice arrangement in the case of MS6 compared to pure excipients was evidenced by the changes in the positions of the melting peak (lowering of the melting enthalpies) of both TOL and Span 60 together with the appearance of a broad endothermic peak at 91.38 °C [20]. It could also be related to the effect of Tween 80 as an edge activator in perturbing the packing characteristics of Span 60 and consequently fluidizing the vesicular bilayer [56]. Hence, the aforementioned results confirmed that TOL dispersed homogenously within spanlastics in an amorphous form.

### 3.8. Ex Vivo Corneal Permeation

**Figure 8:**
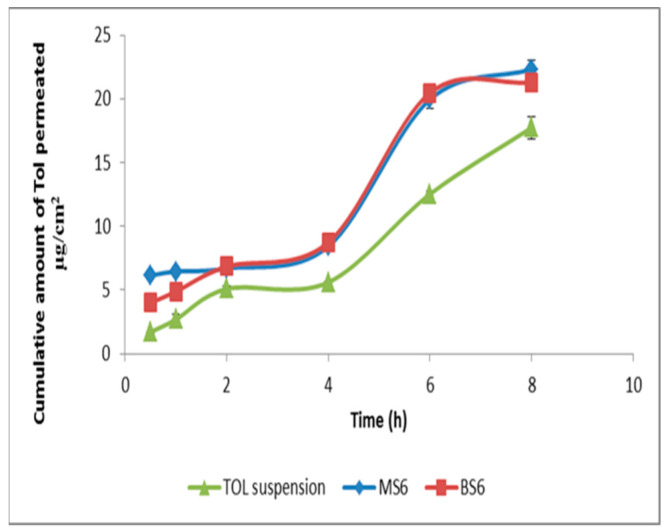
Ex vivo corneal permeation profile of TOL-cosolvent spanlastics (MS6) and basic spanlastics (BS6) compared to TOL suspension.

TOL permeability from MS6 was examined using excised rabbits’ corneas and its corneal permeation profile was constructed and compared to that of BS6 and drug suspension (Figure 8). By comparing the corneal permeability parameters, it was shown that both MS6 and BS6 significantly enhanced the drug flux and resulted in a higher amount of TOL permeated in 8 h compared to TOL suspension (*p* ˂ 0.05) (Table 7). This significantly enhanced TOL ocular delivery through cosolvent-modified spanlastics as well as basic spanlastics could be related to the hydrophilic ingredients of these elastic vesicles (edge activators and propylene glycol) which are attracted preferentially to the area of high water content such as the aqueous humor and the vitreous of the eye which constitutes a majority of water (≈90%). Hence, edge activators enhance the vesicular deep migration in the water-rich environment, carrying drug molecules to secure and acceptable hydration conditions [25,57]. In addition, the nanometric size of these elastic vesicles eased their passage through the narrow hydrated corneal stromal network as a fine dispersion compared to coarse PS of drug suspension [58].

### 3.9. In Vitro Antifungal Activity

#### 3.9.1. Minimum Inhibitory Concentration (MIC)

The microbroth dilution technique was applied in the antifungal assays in order to investigate whether the optimal TOL-cosolvent spanlastics (MS6) impacts the antifungal activity of TOL. The MIC of MS6 (treatment A) was 0.49 µg/mL, while TOL suspension (treatment B) had a higher MIC (1.95 µg/mL) and that for the placebo solution was 125 µg/mL. Hence, the antifungal activity of MS6 was improved in comparison to that of TOL suspension. This might be related to the ability of MS6 to enhance TOL solubility, which consequently enhanced TOL penetration through the fungal cell wall and inhibited ergosterol biosynthesis which is responsible for its activity [16,30].

#### 3.9.2. Minimum Fungicidal Concentration (MFC)

The broth microdilution technique was used to determine MFC for treatment A and B. Both MS6 and TOL suspension exhibited fungicidal effect after 48 h of incubation at 28 °C ± 2 for. MS6 (treatment A) showed a more powerful fungicidal effect at 3.9 µg/mL (8× MIC), whereases MFC of TOL suspension (treatment B) was higher with a value of 125 µg/mL (64× MIC). Therefore, TOL suspension had inferior fungicidal activity compared to that of MS6. 

#### 3.9.3. Kill Kinetics Assay

**Figure 9:**
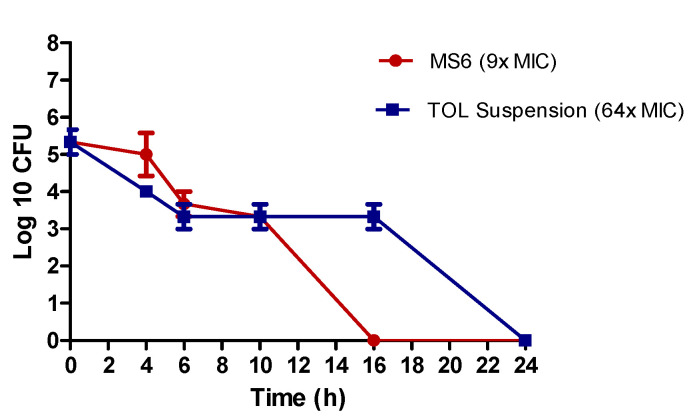
The killing kinetics of treatment A (MS6) and treatment B (TOL suspension) tested against *Aspergillus niger* (ATCC32656). Treatment A tested at concentration of (3.9 µg/mL) (9× MIC), treatment B (TOL suspension) tested at concentration of (125 µg/mL) (64× MIC). Data are represented by means of the number of recovered colonies counted at each time point ± SD, n = 3. The chart was generated using GraphPad Prism (v5) (GraphPad, California, CA, USA).

The killing kinetics of *Aspergillus niger* (ATCC32656) by treatment A and treatment B were investigated. Treatment A (MS6) killed *Aspergillus niger* (ATCC32656) after 16 h incubation at its MFC concentration (3.9 µg/mL) (9× MIC), while longer time (24 h) was needed for treatment B (TOL suspension) to kill *Aspergillus niger* (ATCC32656) at its MFC concentration (125 µg/mL) (64× MIC) (Figure 9). This confirmed the superior anti-aspergillus activity of TOL-cosolvent spanlastics compared to the drug suspension.

### 3.10. In Vivo Studies

#### 3.10.1. Draize Test

The obtained results of the Draize test showed that corneas treated with MS6 did not show irritation, lacrimation, or any sign of inflation compared to the control eye at all tested time points. Hence, they scored zero on the Draize scale. These results suggested the ocular tolerance of the optimally modified spanlastics. 

#### 3.10.2. Histopathology

**Figure 10:**
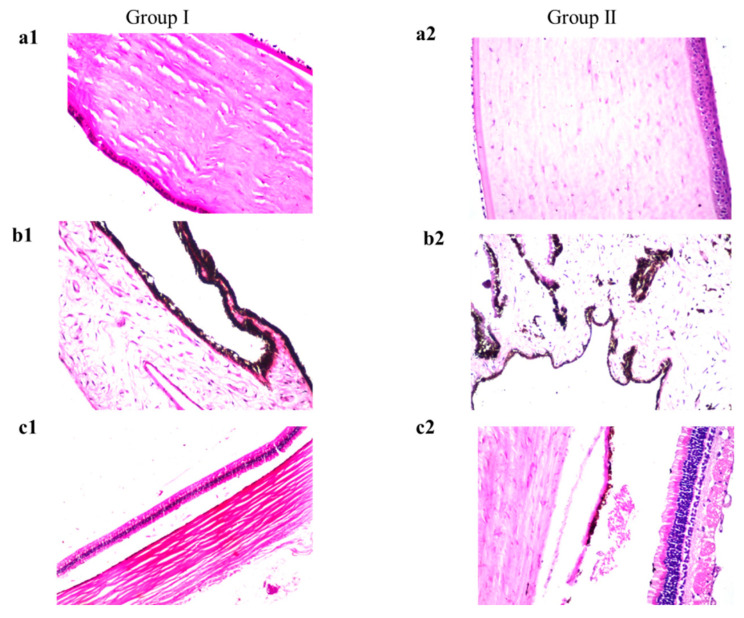
Histopathological photomicrographs (stained with hematoxylin and eosin) showing control rabbit eye (group I) and MS6-treated eye (group II); (**a1**,**a2**) show the cornea, (**b1**,**b2**) show the iris, (**c1**,**c2**) demonstrate the retina, choroid and sclera with normal histological structure (×40 magnification power). Photomicrographs of Group I ((**a1**,**b1,c1**)) were adopted from Aziz et al. [14].

The obtained photomicrographs exhibited the absence of any histopathological changes in the cornea (the lining corneal epithelium, the underlying stroma, and endothelium) (Figure 10(a2)). The same results were observed in the iris (Figure 10(b2)), retina, choroid, and sclera (Figure 10(c2)) compared to control (Figure 10(a1–c1)). The absence of any histopathological abnormalities in ocular tissues after treatment with TOL-cosolvent spanlastics confirmed its tolerability and safety for ocular application.

#### 3.10.3. Susceptibility Test

**Figure 11:**
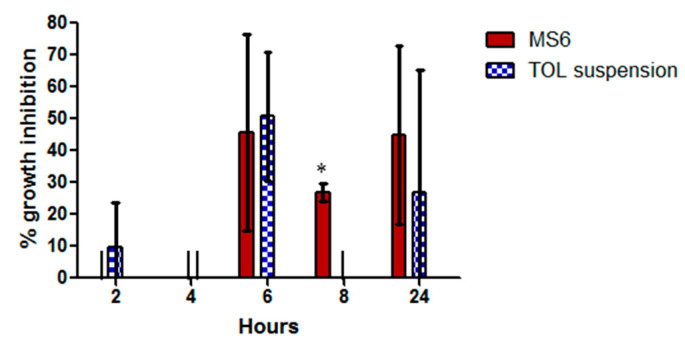
In vivo percentage growth inhibition of MS6 (treatment A) compared to TOL suspension (treatment B) on *Aspergillus niger* (ATCC32656). * Indicates statistically significant difference between the columns applying one-way ANOVA test (*p* < 0.05, n = 3).

The retention time of the drug on the surface of eye following ocular administration strongly affects the percentage growth inhibition of *Aspergillus niger* (Figure 11). The percentage inhibition of MS6 was maximum (45.7 ± 33.66%) after 6 h of administration. Similarly, after 6 h, the TOL suspension reached its maximum inhibition (50.75 ± 20.15%) but then it decreased to 0% inhibition after 8 h and 27 ± 38.18% after 24 h. The percentage growth inhibition of MS6 was significantly greater than that of TOL suspension after 8 h (*p* = 0.005) of administration (one-way ANOVA, *p* < 0.05). 

TOL-cosolvent spanlastics had prolonged antifungal activity in the eye compared to TOL suspension. The area under the curve for MS6 was 1.5 folds greater than that of TOL suspension (AUC_2 h–24 h_ = 458.6 and 300.3, respectively). This might be related to the PS of MS6 which was smaller than that of drug suspension which consequently increased the residence of TOL on the cornea [15,20]. In addition, the components of the modified spanlastics formula such as the cosolvent and edge activators enhanced the corneal penetration of TOL. Therefore, MS6 is an encouraging substitute for eye drops due to its prolonged antifungal effect.

## 4. Conclusions

In this work, TOL basic spanlastics were formulated using the ethanol injection method according to a full 3^2^ factorial design to analyze the formulation variable's effect on the characteristics of TOL spanlastics characteristics and to select the optimal formulation for further modification. BS6 was selected as the nucleus for developing novel TOL-modified spanlastics by incorporating different cosolvent in different concentrations in their constructs. TOL-modified spanlastics were prepared using the same procedures of preparing basic spanlastics according to 3^1^2^1^ full factorial designs. MS6, prepared using 30% propylene glycol, was the optimized TOL-cosolvent spanlastics with a desirability value of 0.895. MS6 was compared to BS6 and both of them presented superior corneal permeation potential compared to a drug suspension. The safety and tolerability of MS6 were confirmed by the in vivo histopathological studies. The fungal susceptibility of *Aspergillus niger* (ATCC32656) to TOL using confirmed the superior residence of MS6 in the cornea compared to a drug suspension. Concisely, it can be concluded that modified spanlastics can be an effective ocular treatment for *Aspergillus niger-*induced fungal keratitis.

## Figures and Tables

**Table 1:** Full 3^2^ factorial design for TOL basic spanlastics optimization.

**Factors**	**Levels**		
X_1_: Span 60 amount (mg)	200	300	400
X_2_: Tween 80 amount (mg)	50	100	150
**Responses (dependent variables)**	**Desirability Constraints**		
Y_1_: EE%	Maximize		
Y_2_: PS (nm)	Minimize		
Y_3_: ZP (mV)	Maximize (as absolute value)		

Abbreviations: EE%, entrapment efficiency; PS, particle size; ZP, zeta potential.

**Table 2:** Full (3^1^.2^1^) factorial design for optimization of TOL-cosolvent spanlastics.

**Factors**	**Levels**			
X_1_: Type of cosolvent	Glycerol		Propylene glycol	
X_2_: Percentage of cosolvent	10	20		30
**Responses (dependent variables)**	**Desirability Constraints**			
Y_1_: EE%	Maximize			
Y_2_: PS (nm)	Minimize			
Y_3_: ZP (mV)	Maximize (as absolute value)			

Abbreviations: EE%, entrapment efficiency percent; PS, particle size; ZP, zeta potential.

**Table 3:** Experimental runs, independent variables, and measured response of 3^2^ full factorial experimental designs of TOL basic spanlastics.

RunsRuns	X1	X_2_	Y_1_	Y_2_	Y_3_
	Span 60 Amount (mg)	Tween 80 Amount (mg)	EE % ^a^	PS (nm) ^a^	ZP (mV) ^a^
BS1	200	50	27.85 ± 1.20	238 ± 1.98	−23.75 ± 1.20
BS2	300	50	32.05 ± 3.89	271.8 ± 11.03	−28.35 ± 0.78
BS3	400	50	51.15 ± 0.49	436.55 ± 9.97	−39.45 ± 0.92
BS4	200	100	31.05 ± 1.63	349.55 ± 34.44	−23.25 ± 0.78
BS5	300	100	49.8 ± 12.30	300.14 ± 0.14	−29.75 ± 1.20
BS6	400	100	76.8 ± 7.07	282.75 ± 1.34	−28.75 ± 2.76
BS7	200	150	30.9 ± 2.26	231.5 ± 10.18	−24.85 ± 0.64
BS8	300	150	47.3 ± 2.12	330.55 ± 44.48	−31.85 ± 0.78
BS9	400	150	85.45 ± 1.77	400.40 ± 6.51	−32.95 ± 1.48

Abbreviations: EE%, entrapment efficiency; PS, particle size; ZP, zeta potential; BS, basic spanlastics. ^a^ Data represented as mean ± SD (n = 3).

**Table 4:** Output data of the full factorial analysis of TOL basic spanlastics.

Responses	R^2^	Adjusted R^2^	Predicted R^2^	Adequate Precision	Significant Factors
EE%	0.9676	0.9388	0.8703	15.990	X_1_ (<0.0001), X_2_ (=0.0004)
PS (nm)	0.9602	0.9249	0.8409	14.628	X_1_ (<0.0001)
ZP (mV)	0.9641	0.9321	0.8562	17.310	X_1_ (<0.0001), X_2_(=0.0047)

Abbreviations: EE%, entrapment efficiency; PS, particle size; ZP, zeta potential.

**Table 5:** Experimental runs, independent variables, and measured responses of 3^1^.2^1^ full factorial experimental design of TOL-cosolvent spanlastics.

Runs	X_1_	X_2_	Y_1_	Y_2_	Y_3_
	Cosolvent Type	Cosolvent Percentage	EE% ^a^	PS (nm) ^a^	ZP (mV) ^a^
MS1	Glycerol	10	71 ± 5.66	416.5 ± 33.23	−30.9 ± 1.13
MS2	Glycerol	20	37.8 ± 1.56	285.5 ± 11.17	−29.75 ± 2.90
MS3	Glycerol	30	72.5 ± 2.83	581.5 ± 2.55	−31.9 ± 1.13
MS4	Propylene glycol	10	56.73 ± 15.09	328.55 ± 14.21	−31.8 ± 0.71
MS5	Propylene glycol	20	57.45 ± 2.76	374.85 ± 32.74	−28.7 ± 2.12
MS6	Propylene glycol	30	66.1 ± 0.57	231.2 ± 0.141	−32.15 ± 0.07

Abbreviations: EE%, entrapment efficiency percent; PS, particle size ZP, zeta potential; MS, modified spanlastics. ^a^ Data represented as mean ± SD (n = 3).

**Table 6:** Output data of the full factorial analysis of TOL-cosolvent spanlastics.

Responses	R^2^	Adjusted R^2^	Predicted R^2^	Adequate Precision	Significant Factors
EE%	0.8557	0.7354	0.4226	7.209	X_2_ (=0.0089)
PS (nm)	0.9836	0.9699	0.9342	24.226	X_1_ (<0.0001), X_2_ (=0.0009)
ZP (mV)	0.5441	0.1641	−0.8238	2.991	---------

Abbreviations: EE%, entrapment efficiency percent; PS, particle size; ZP, zeta potential.

**Table 7:** Ex vivo corneal permeability parameters of TOL-cosolvent spanlastics (MS6) and basic spanlastics (BS6) compared to a drug suspension.

Formulation	Total Amount of TOL Permeated per Unit Area in 8 h (µg/cm^2^) ^a^	J_max_ (µg/cm^2^/h) ^a^	ER
MS6	22.33 ± 0.67	4.36 ± 0.13	1.26
BS6	21.30 ± 0.07	4.16 ± 0.01	1.20
Drug suspension	17.74 ± 0.89	3.46 ± 0.17	1

Abbreviations: J_max_, flux; ER, enhancement ratio; MS, modified spanlastics; BS, basic spanlastics. ^a^ Data presented as mean ± SD (n = 3).

## Data Availability

Not applicable.
